# Balloon-expandable transcatheter aortic valve implantation using the cusp-overlap technique for accurate valve positioning

**DOI:** 10.1093/ehjcr/ytae087

**Published:** 2024-02-21

**Authors:** Masaki Tsuda, Yasuyuki Egami, Shodai Kawanami, Masami Nishino

**Affiliations:** Division of Cardiology, Osaka Rosai Hospital, 1179-3 Nagasone-cho, Sakai, 591-8025 Osaka, Japan; Division of Cardiology, Osaka Rosai Hospital, 1179-3 Nagasone-cho, Sakai, 591-8025 Osaka, Japan; Division of Cardiology, Osaka Rosai Hospital, 1179-3 Nagasone-cho, Sakai, 591-8025 Osaka, Japan; Division of Cardiology, Osaka Rosai Hospital, 1179-3 Nagasone-cho, Sakai, 591-8025 Osaka, Japan

## Case description

An 80-year-old woman presented to our hospital with exertional dyspnoea. Transthoracic echocardiography demonstrated preserved left ventricular ejection fraction (73%) and severe aortic stenosis (mean pressure gradient, 43 mmHg). The patient had a moderate surgical risk and suitable anatomy for transfemoral transcatheter aortic valve implantation (TAVI). Pre-procedural computed tomography revealed an annulus area of 395 mm^2^ with a short membranous septum (1.3 mm); therefore, we planned transfemoral TAVI using a 23 mm SAPIEN 3 valve (S3; Edwards Lifesciences, Irvine, CA) with high positioning to avoid pacemaker implantation.

**Figure 1:**
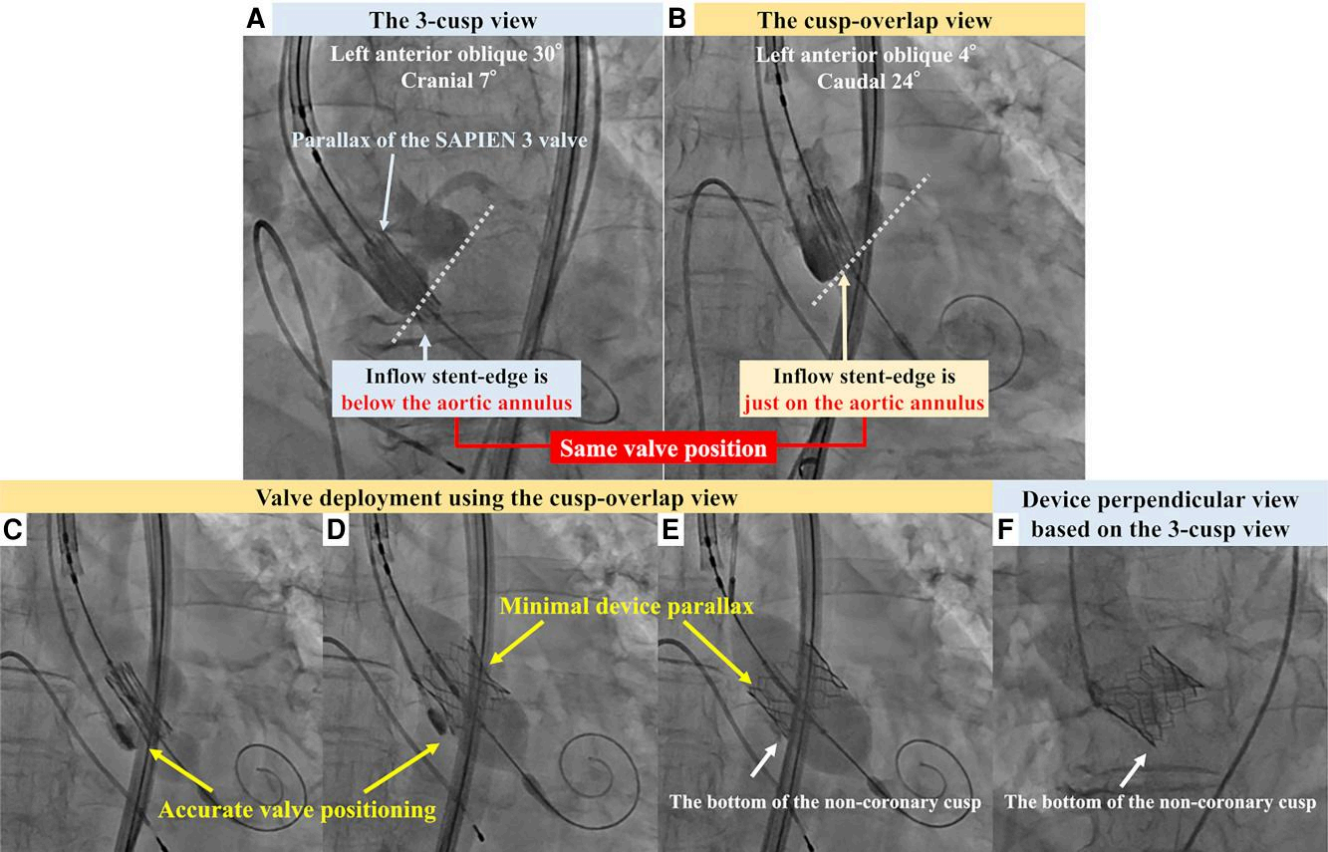
Aortography before valve deployment (upper row). (*A*) The three-cusp and (*B*) cusp-overlap views with the same valve position. The dotted lines show the aortic annulus in each view. Fluoroscopic images during valve deployment with the cusp-overlap view (lower row). (*C*) Half-, (*D*) two-third-, and (*E*) full-balloon inflation. A pigtail catheter being inserted in the non-coronary cusp. (*F*) Final aortography.

After advancing the S3 through the aortic valve, the radiolucent line of the S3 was aligned with the aortic annulus in the three-cusp view. However, the S3 parallax made accurate positioning difficult (*Figure 1A*; Supplementary material online, *Video S1*); the cusp-overlap (CO) view avoided the S3 parallax when used in the same valve position. Furthermore, aortography in the CO view demonstrated that the inflow stent edge was positioned precisely on the aortic annulus, even though it appeared to be located on the ventricular side in the three-cusp view (*Figure 1B*; Supplementary material online, *Video S2*). After repositioning the valve towards the ventricular side, the S3 was implanted using the CO view entirely under rapid pacing (*Figure 1C–E*; Supplementary material online, *Video S3*), which enabled valve deployment without device parallax. Aortography after implantation showed that the S3 was implanted in the intended position without significant paravalvular leakage (*Figure 1F*; Supplementary material online, *Video S4*).

Advantages of the CO technique, including the elimination of device parallax, have been suggested previously, but this technique has rarely been performed in practice during the deployment of balloon-expandable valves.^1^ This case demonstrates the utility of the CO technique in accurately positioning the valve, avoiding valve malpositioning and embolization. The CO technique is useful in TAVI using balloon-expandable valves, especially when a valve needs to be implanted at a high level to avoid pacemaker implantation.

## Supplementary Material

ytae087_Supplementary_Data

## Data Availability

The data underlying this article are available in the article and in its online Supplementary material.

## Supplementary material

**Consent:** The authors confirm that written consent for the submission and publication of this case report, including the accompanying images and associated text, has been obtained from the patient in line with the COPE guidelines.

**Funding:** None declared.
